# Polymyalgia rheumatica in der 18-Fluordesoxyglukose-Positronenemissionstomographie/Computertomographie: Verbesserung diagnostischer Genauigkeit und Abgrenzung von rheumatoider Arthritis

**DOI:** 10.1007/s00393-021-01133-w

**Published:** 2021-12-01

**Authors:** F. Witte, H.-J. Lakomek, J. Holzinger, W.-D. Reinbold

**Affiliations:** 1 Johannes Wesling Klinikum Minden, Universitätsklinikum der Ruhr-Universität Bochum, Minden, Deutschland; 2 Universitätsklinik für Geriatrie, Mühlenkreiskliniken, Johannes Wesling Klinikum Minden, Minden, Deutschland; 3 Universitätsinstitut für Radiologie, Neuroradiologie und Nuklearmedizin, Mühlenkreiskliniken, Johannes Wesling Klinikum Minden, Minden, Deutschland

**Keywords:** Entzündung, Glukokortikoide, Diagnose, Rheumatoide Arthritis, Riesenzellarteriitis, Inflammation, Glucocorticoids, Diagnosis, Rheumatoid arthritis, Giant cell arteritis

## Abstract

**Hintergrund:**

Die Diagnose von Patienten mit Polymyalgia rheumatica (PMR) beruht bislang auf der klinischen Symptomatik und laborchemischen Entzündungsparametern. Aktuell wird der Nutzen verschiedener bildgebender Verfahren evaluiert, hierunter die Sonographie, MRT und PET.

**Ziel der Arbeit/Fragestellung:**

Ziel war die Evaluation der diagnostischen Wertigkeit der 18-Fluordesoxyglukose-Positronenemissionstomographie/Computertomographie (18F-FDG-PET/CT) bei PMR, um die Sensitivität und Spezifität in der diagnostischen Aufarbeitung zu verbessern, sowie die rheumatoide Arthritis (RA) differentialdiagnostisch verbessert abzugrenzen.

**Material und Methoden:**

Es wurden 18F-FDG-PET/CT-Untersuchungen von 284 rheumatologischen Patienten – hierunter 97 Patienten mit PMR – aus einem 44-monatigen Zeitraum retrospektiv evaluiert. Weiter wurden 13 entzündlich veränderte Regionen via dreidimensionaler Region-of-interest(ROI)-Messung mit Bestimmung des maximalen Standardized-Uptake-Value (SUVmax) analysiert, gefolgt von statistischen Analysen.

**Ergebnisse und Diskussion:**

Patienten mit PMR zeigten im Vergleich mit einer rheumatologisch behandelten Kontrollgruppe signifikant erhöhte Anreicherungen in allen gemessenen Regionen (*p* < 0,001). Die Methode mit der stärksten diagnostischen Aussagekraft stellte die Kombination aus vier SUVmax-Messwerten – beider anterolateraler Hüftkapseln und beider Tubera ischiadica – dar, mit einer Sensitivität von 91,3 % und einer Spezifität von 97,6 % bei einem Cut-off von 11,0 SUV für die Erstdiagnose von PMR-Patienten, die noch keine immunsuppressive Therapie erhalten hatten. Patienten mit RA konnten bei Erstdiagnose an ebenjenen anatomischen Regionen signifikant von Patienten mit PMR unterschieden werden (*p* < 0,001).

## Abstract (en)

**Background:**

The diagnosis of patients with polymyalgia rheumatica (PMR) has relied upon the clinical examination of symptoms and laboratory parameters of inflammation until now. Currently, the use of different imaging modalities is being explored, including ultrasound, MRI and PET.

**Objectives:**

The aim was to evaluate the diagnostic value of 18-fluorodeoxyglucose-positron-emission-tomography/computed tomography (18F-FDG-PET/CT) for PMR, in order to improve the sensitivity and specificity of diagnosing PMR and to improve the differential diagnosis of rheumatoid arthritis (RA).

**Materials and Methods:**

Examinations using 18F-FDG-PET/CT of 284 rheumatological patients, including 97 patients with PMR, were retrospectively evaluated over a 44-month period. Furthermore, 13 regions changed by inflammation were analysed via a three-dimensional region of interest (ROI) measurement with determination of maximum standardized uptake values (SUVmax), followed by statistical analyses.

**Results and Discussion:**

Patients with PMR presented significantly elevated uptake in all regions examined (*p* < 0.001), compared with a control group treated for rheumatological diseases. The method with the highest diagnostic relevance was represented by the combination of four SUVmax values of both anterolateral hip capsules and both ischial tuberosities, reaching a sensitivity of 91.3% and a specificity of 97.6% with a cut-off of 11.0 SUV at the initial diagnosis of PMR patients who had not yet received any immunosuppressive therapy. Patients with RA could be significantly distinguished from those with PMR at initial diagnosis in the same anatomical regions (*p* < 0.001).

## Hintergrund

Für die klinische Diagnostik von Patienten mit Polymyalgia rheumatica (PMR) existiert kein spezifischer Laborparameter [1, 18, 22, 24, 30] und weitere rheumatologische Krankheitsbilder mit ähnlicher Symptomatik, wie rheumatoide Arthritis (RA) und Spondylarthropathien (SPA), erschweren die differentialdiagnostische Abgrenzung. Die Bedeutung der 18-Fluordesoxyglukose-Positronenemissionstomographie/Computertomographie(18F-FDG-PET/CT) wird zunehmend diskutiert [12, 20, 21], aktuell vor allem die Kombination spezifischer anatomischer Messregionen [13, 27]. Ein Zusatznutzen dieser Untersuchungsmethode bei Patienten mit PMR besteht im Ausschluss einer Riesenzellarteriitis (RZA) sowie eines Malignoms [16, 21].

## Patienten und Methoden

### Patienten

Die Ganzkörper-18F-FDG-PET/CT-Untersuchungen von 284 Patienten, 150 Frauen und 134 Männern mit einem Alters-Median von 64,5 Jahren, die zwischen dem 15.01.2014 und dem 06.09.2017 in der Universitätsklinik für Rheumatologie und Geriatrie des Universitätsklinikums Minden durchgeführt worden sind, wurden retrospektiv ausgewertet. Alle untersuchten Patienten hatten eine zugrunde liegende rheumatische Erkrankung oder den Verdacht auf eine solche und befanden sich zum Zeitpunkt der Untersuchung in stationärer rheumatologischer Behandlung. In der Fallgruppe hatten 97 Patienten (34 %) eine PMR, davon 9 (9 %) mit einer begleitenden Riesenzellarteriitis (RZA). In der Kontrollgruppe hatten 39 Patienten eine RA sowie 17 eine Spondylarthropathie wie Psoriasisarthritis und Morbus Bechterew, 10 eine Granulomatose mit Polyangiitis, 9 eine RZA und 8 einen Morbus Still. Bei 38 % der RZA-Patienten bestand gleichzeitig eine PMR.

Die klinische Diagnose erfolgte basierend auf Expertenmeinungen, die sich in Bezug auf PMR an den klinischen Kriterien von Bird und denjenigen der EULAR/ACR-Initiative orientierten.

### Methoden

Die 18F-FDG-PET/CT-Untersuchungen mittels PET/CT-Scanner (Philips Gemini GXL 6, Philips Medizin Systeme, Böblingen, Deutschland, 2008) erfolgten nach Gabe des Tracers (Aktivität 3 MBq/kgKG [Megabecquerel pro Kilogramm Körpergewicht], mit einer maximalen Aktivität von 300 MBq, effektiven Äquivalenzdosis von 0,02 mSv/MBq [Millisievert pro Megabecquerel] entsprechend ca. 4,2 mSv pro Untersuchung bei einem 70 kg schweren Patienten) nach einer Akkumulationszeit von 45–120 min. Die Messung der Anreicherungen in den 18F-FDG-PET/CTs erfolgte per dreidimensionaler Region-of-Interest(3D-ROI)-Messung und mittels maximalem Standardized-Uptake-Value (SUVmax) an nachfolgenden Lokalisationen:

- Beidseitig:
  - gesamte Hüfte
  - Trochanter major
  - anterolaterale Hüftkapsel
  - gesamte Schulter
  - anteromediales Schultergelenk
  - Tuber ischiadicum
- Einfach:
  - interspinal/spinaler Raum der Processus spinosi der Lendenwirbelsäule

Folgende Gruppen des Patientenkollektivs wurden im statistischen Vergleich der SUVmax-Werte untersucht:

Fallgruppe:

- PMR-Gruppe allgemein
- Vier PMR-Subgruppen:
  - Erstdiagnose PMR ohne immunsuppressive Therapie
  - Erstdiagnose PMR mit immunsuppressiver Medikation in Form eines Steroids im stationären Aufenthalt unmittelbar vor der 18F-FDG-PET/CT
  - Erstdiagnose PMR mit immunsuppressiver Medikation im Zeitraum vor der stationären Aufnahme
  - im Vorfeld der stationären Aufnahme gestellte PMR-Diagnose mit bereits erfolgter immunsuppressiver Therapie

Kontrollgruppe (übriges Patientenkollektiv, nicht PMR):

- Kontrollgruppe allgemein
- RA-Gruppe (Teil der Kontrollgruppe)
  - Erstdiagnose RA
  - bereits gestellte Diagnose RA

Die SUVmax-Werte aller gemessenen Lokalisationen von PMR-Patienten und diejenigen von Nicht-PMR-Patienten wurden mittels unabhängigem t‑Test auf einen signifikanten Unterschied hin evaluiert. Durch Auftreten einer Kumulation der Alpha-Fehler-Wahrscheinlichkeit (Fehler 1. Art) bei multiplen Tests in derselben Stichprobe wurde zusätzlich per Bonferroni-Korrektur für multiple Vergleiche korrigiert.

Mit der gleichen Methode wurden PMR-Patienten mit RA-Patienten verglichen. Zusätzlich erfolgte hier eine Subgruppenanalyse. Der Vergleich der erstdiagnostizierten PMR-Patienten mit der Gruppe der erstdiagnostizierten RA-Patienten erfolgte aufgrund der kleinen Gruppengröße mittels Mann-Whitney-U-Test für unabhängige Stichproben.

Abschließend wurden die Sensitivität und die Spezifität der SUVmax-Messung in der 18F-FDG-PET/CT für die verschiedenen PMR-Subgruppen statistisch untersucht. Es wurde bei symmetrischen Messorten die Summe beider Regionen gebildet. Zur optimalen Grenzziehung wurde der Cut-off-Wert über die Berechnung der Receiver-Operating-Characteristic (ROC)-Kurven und die größte Area under the curve (AUC) bestimmt. Cut-off-Werte stellen SUVmax-Werte dar.

Für die Signifikanztests wurde ein Signifikanzniveau von *p* < 0,05 festgelegt.

### Ausschlusskriterien

Aufgrund von extremen, durch Hüftimplantate sowie starke Knochenmarkaktivierung und Metastasen im Messbereich hervorgerufenen Messwerten, die die SUVmax-Messung dominierten, konnten keine validen Messdaten erhoben werden. Dies betraf drei Patienten der Fall- und 23 Patienten der Kontrollgruppe, davon ein RA-Patient.

## Ergebnisse

### Polymyalgia rheumatica in der 18F-FDG-PET/CT

**Figure 1:**
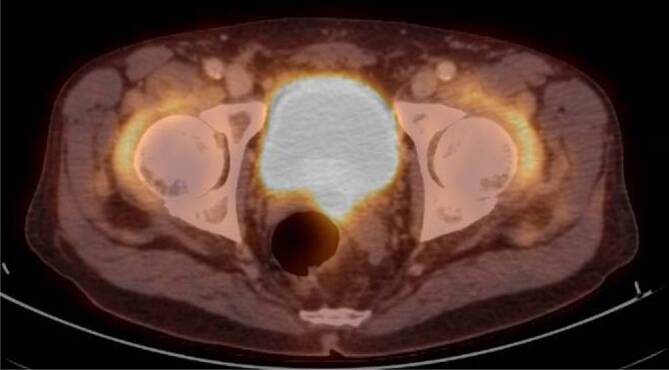

Patienten der Fallgruppe mit PMR zeigten auch nach Bonferroni-Korrektur einen signifikant höheren Messwert des SUVmax als nicht an PMR erkrankte Patienten der Kontrollgruppe (*p* < 0,001) (Tab. 1) – hierunter auch Patienten mit RA und SPA – an allen untersuchten Regionen. Die Anreicherungen projizierten sich auf die großen Gelenke des Körperstammes, die Sitzbeine (Abb. 1 und 2), sowie die interspinal lumbale Region.

**Table 1:** 

SUV-Messregion	PMR vs. nicht PMR	*N*	Mittelwert	Std.-Abweichung	Statistik
Anterolaterale Hüftkapsel rechts	PMR	94	3,087	1,5157	t_(128,35)_ = 8,96, *p* < 0,001
	Nicht PMR	164	1,561	0,8643	
Anterolaterale Hüftkapsel links	PMR	94	2,845	1,4856	t_(105,89)_ = 8,74, *p* < 0,001
	Nicht PMR	164	1,461	0,5134	
Summe anterolaterale Hüftkapseln	PMR	94	5,932	2,8369	t_(115,76)_ = 9,4, *p* < 0,001
	Nicht PMR	164	3,022	1,2995	
Tuber ischiadicum rechts	PMR	94	2,744	1,2106	t_(117,44)_ = 9,24, *p* < 0,001
	Nicht PMR	164	1,518	0,5745	
Tuber ischiadicum links	PMR	94	2,686	1,2071	t_(149,68)_ = 8,02, *p* < 0,001
	Nicht PMR	164	1,548	0,8746	
Summe Tubera ischiadica	PMR	94	5,430	2,3701	t_(123,36)_ = 8,98, *p* < 0,001
	Nicht PMR	164	3,065	1,2527

**Figure 2:**
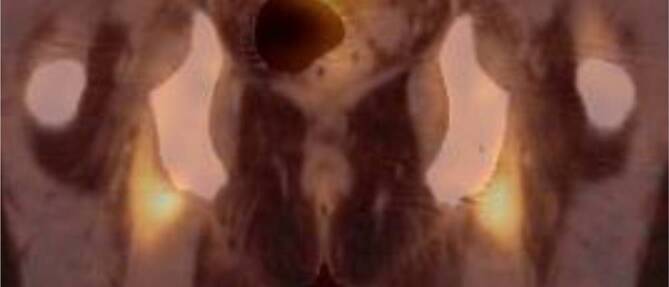

Hinsichtlich der Diagnostik von Patienten mit PMR via 18F-FDG-PET/CT zeigte sich, dass die Kombination der SUVmax-Messwerte beider anterolateraler Hüftkapseln und beider Tubera ischiadica am besten geeignet ist, Patienten mit PMR zu identifizieren. Im Gruppenvergleich aller PMR-Patienten (*n* = 94) gegenüber allen Nicht-PMR-Patienten (*n* = 164) errechnete sich ein Cut-off-Wert von 7,3 SUV (Sensitivität 0,745, Spezifität 0,829).

### Polymyalgia rheumatica – Subgruppenanalyse bezüglich immunsuppressiver Medikation und Zeitpunkt der Diagnose

#### PMR – Erstdiagnose ohne Vortherapie

**Figure 3:**
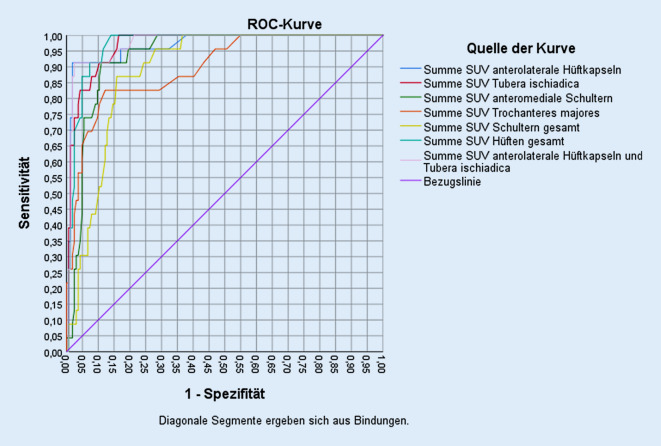

Optimale Ergebnisse ergab die PMR-Subgruppe, die zum Zeitpunkt der 18F-FDG-PET/CT-Untersuchung noch unter keiner immunsuppressiven Therapie stand und erstdiagnostiziert wurde. Im Vergleich dieser Subgruppe der PMR-Patienten mit der Nicht-PMR Gruppe lag der Cut-off-Wert bei 11,0 SUV (Sensitivität 0,913, Spezifität 0,976) (Tab. 2; Abb. 3). Der negative prädiktive Wert erreichte 98,8 % bei einem positiven prädiktiven Wert von 84 %.

**Table 2:** 

	*N*
PMR ED ohne Vortherapie	23
Nicht PMR	164

#### PMR – Erstdiagnose mit stationärer Steroidtherapie

Der Vergleich der Subgruppe der PMR-Patienten bei Erstdiagnose und einer stationär begonnenen Steroidtherapie (*n* = 19) mit der Kontrollgruppe ergab einen Cut-off-Wert von 8,6 SUV (Sensitivität 0,789, Spezifität 0,927).

#### PMR – Erstdiagnose mit prästationärer Immunsuppression

Im Vergleich der Gruppe der PMR-Patienten bei Erstdiagnose und bereits prästationär applizierter immunsuppressiver Therapie (*n* = 13) mit der Kontrollgruppe zeigte sich ein Cut-off-Wert von 8,45 SUV (Sensitivität 0,923, Spezifität 0,921).

#### PMR – vorbekannt

Der Vergleich der PMR-Patienten bei bereits bekannter Diagnose (*n* = 30) mit der Kontrollgruppe lieferte einen Cut-off-Wert von 6,35 SUV (Sensitivität 0,767, Spezifität 0,689).

### Differentialdiagnostik Polymyalgia rheumatica und rheumatoide Arthritis

#### Gesamtvergleich PMR vs. RA

Im Vergleich der untersuchten Regionen zwischen PMR-Patienten und RA-Patienten zeigte sich ein signifikant höherer SUVmax-Messwert der PMR-Patienten in der Summe der gesamten Hüftregion, insbesondere der anterolateralen Hüftkapsel, den Tubera ischiadica (Tab. 3), den Trochanteres majores sowie interspinal lumbal. Es konnte kein signifikanter Unterschied in der Summe der gesamten Schulter, insbesondere der anteromedialen Gelenkseite, dem Ansatz des M. subscapularis entsprechend, festgestellt werden.

**Table 3:** 

SUV-Messregion	PMR vs. alle RA	*N*	Mittelwert	Std.-Abweichung	Signifikanz
Anterolaterale Hüftkapsel rechts	PMR	94	3,087	1,5157	t_(127,2)_ = 7,92, *p* < 0,001
	RA	38	1,550	0,7101	
Anterolaterale Hüftkapsel links	PMR	94	2,845	1,4856	t_(129,48)_ = 7,07, *p* < 0,001
	RA	38	1,537	0,6377	
Summe anterolaterale Hüftkapseln	PMR	94	5,932	2,8369	t_(127,79)_ = 7,88, *p* < 0,001
	RA	38	3,087	1,3062	
Tuber ischiadicum rechts	PMR	94	2,744	1,2106	t_(120,76)_ = 6,97, *p* < 0,001
	RA	38	1,611	0,6409	
Tuber ischiadicum links	PMR	94	2,686	1,2071	t_(130)_ = 3,17, *p* = 0,002
	RA	38	1,879	1,5883	
Summe Tubera ischiadica	PMR	94	5,430	2,3701	t_(83,31)_ = 4,88, *p* < 0,001
	RA	38	3,489	1,9347	

#### Erstdiagnose PMR vs. Erstdiagnose RA

Differenzierte man beide Patientengruppen genauer in diejenigen mit einer Erstdiagnose und diejenigen mit bereits länger bekannter Erkrankung, so zeigten sich in der Gruppe der Erstdiagnose signifikant höhere Messwerte in der PMR-Gruppe in der Summe der gesamten Hüften, der anterolateralen Hüftkapsel und den Tubera ischiadica (Tab. 4).

**Table 4:** 

SUV-Messregion	ED PMR vs. ED RA	*N*	Mittelwert	Std.-Abweichung	Signifikanz
Anterolaterale Hüftkapsel rechts	ED PMR	55	3,785	1,4373	Z = 3,69, *p* < 0,001
	ED RA	7	1,529	0,5648	
Anterolaterale Hüftkapsel links	ED PMR	55	3,435	1,4656	Z = 3,31, *p* < 0,001
	ED RA	7	1,586	0,4706	
Summe anterolaterale Hüftkapseln	ED PMR	55	7,220	2,6755	Z = 3,58, *p* < 0,001
	ED RA	7	3,114	1,0205	
Tuber ischiadicum rechts	ED PMR	55	3,304	1,1682	Z = 3,42, *p* < 0,001
	ED RA	7	1,643	0,6106	
Tuber ischiadicum links	ED PMR	55	3,200	1,2276	Z = 3,19, *p* = 0,001
	ED RA	7	1,614	0,8649	
Summe Tubera ischiadica	ED PMR	55	6,504	2,3458	Z = 3,27, *p* < 0,001
	ED RA	7	3,257	1,3831	

#### Bekannte PMR vs. bekannte RA

Waren beide Erkrankungen bereits länger bekannt, fanden sich, wenn für multiple Vergleiche korrigiert wurde, keine signifikanten Unterschiede (Tab. 5).

**Table 5:** 

SUV-Messregion	Vorbekannte PMR vs. vorbekannte RA	*N*	Mittelwert	Std.-Abweichung	Signifikanz
Anterolaterale Hüftkapsel rechts	Vorbekannte PMR	30	1,990	0,8310	t_(58)_ = 2,08, *p* = 0,042*
	Vorbekannte RA	30	1,563	0,7582	
Anterolaterale Hüftkapsel links	Vorbekannte PMR	30	1,893	0,9479	*n*. sig
	Vorbekannte RA	30	1,527	0,6873	
Summe anterolaterale Hüftkapseln	Vorbekannte PMR	30	3,883	1,5935	t_(58)_ = 2,05, *p* = 0,045*
	Vorbekannte RA	30	3,090	1,3993	
Tuber ischiadicum rechts	Vorbekannte PMR	30	1,987	0,7162	t_(58)_ = 2,64, *p* = 0,011*
	Vorbekannte RA	30	1,543	0,5758	
Tuber ischiadicum links	Vorbekannte PMR	30	2,003	0,7107	*n*. sig
	Vorbekannte RA	30	1,937	1,7444	
Summe Tubera ischiadica	Vorbekannte PMR	30	3,990	1,3639	*n*. sig
	Vorbekannte RA	30	3,480	2,0594	

*Pcrit (nach Bonferroni-Korrektur) wäre 0,0026: Alle T‑Tests außer die mit * gekennzeichneten sind auch nach Bonferroni-Korrektur für multiple Vergleiche noch signifikant

## Diskussion

### Diagnostische Wertigkeit der 18F-FDG-PET/CT

Die Diagnose der PMR beruht bisher auf einer klinischen Evaluation, die sich an der Beschwerdesymptomatik sowie Laborwerten orientiert. Verschiedene Klassifikationskriterien wurden für die Erkrankung der PMR entwickelt. Die aktuellsten Kriterien stammen aus dem Jahr 2012 von einer Initiative der European League Against Rheumatism/American College of Rheumatology (EULAR/ACR). Sie erwiesen sich in der Summe von Sensitivität und Spezifität gegenüber denjenigen von Bird [2] sowie Jones und Nobunga und Healey und Chuang als überlegen [15]. Sie erreichten eine Sensitivität von 68 % und eine Spezifität von 78 % für die Unterscheidung von PMR- gegenüber Nicht-PMR-Patienten; unter Erweiterung durch eine Ultraschalluntersuchung der Schultern und Hüften verbesserte sich die Spezifität auf 81 % bei einer verringerten Sensitivität von 66 %. Diese Klassifikationskriterien wurden ausdrücklich als provisorisch publiziert und stellen keine diagnostischen Kriterien dar.

Auch die Klassifikationskriterien der EULAR/ACR-Initiative berücksichtigen die 18F-FDG-PET/CT bisher nicht [5]. Ziel dieser Arbeit war es, die Wertigkeit dieser neuen Untersuchungsmethode für die PMR-Diagnostik zu evaluieren.

Bei Patienten mit PMR findet sich allgemein eine Entzündung der Synovia von Schulter- und Hüftgelenken sowie der periartikulären Region [8, 17, 21]. In der 18F-FDG-PET/CT zeigt sich bei Patienten mit PMR in der Regel konkret eine vermehrte Aufnahme radioaktiv markierter Glucose an den Schultern, den Hüften und den Trochanteres majores, an den Tubera ischiadica sowie weniger häufig zwischen den Processus spinosi der Lendenwirbelsäule [3, 12, 19–21, 26]. Verschiedene Arbeiten postulieren eine Erhöhung der diagnostischen Genauigkeit für Patienten mit PMR durch die 18F-FDG-PET/CT-Untersuchung, im Vergleich zu rein klinischen Kriterien [12, 20, 21].

**Figure 4:**
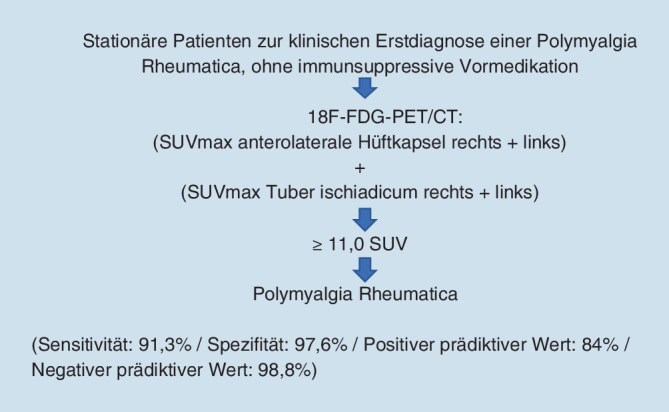

Hinsichtlich der Diagnostik von Patienten mit PMR via 18F-FDG-PET/CT stellte sich im Rahmen unserer Arbeit heraus, dass die Kombination der SUVmax-Messwerte beider anterolateraler Hüftkapseln und beider Tubera ischiadica am besten geeignet ist, Patienten mit PMR zu identifizieren. Insbesondere war diese Kombination in Bezug auf Sensitivität und Spezifität für diejenige PMR-Subgruppe optimal, die zum Zeitpunkt der 18F-FDG-PET/CT-Untersuchung erstdiagnostiziert und noch ohne immunsuppressive Medikation war (Abb. 4).

Unsere Analyse zeigte weiterhin, dass mit steigender Dauer der Immunsuppression vor der 18F-FDG-PET/CT die Spezifität dieser diagnostischen Methode zwar kontinuierlich sinkt, sie allerdings zum Zeitpunkt der Erstdiagnose dieser Erkrankung, vermutlich also im Frühstadium, immer bei über 90 % liegt.

Die Sensitivität der 18F-FDG-PET/CT für die Diagnostik der PMR ist anfällig für eine steroidale Vormedikation. So zeigte sich, dass im Akutstadium eine wenige Tage vor der Untersuchung begonnene Steroidtherapie die Sensitivität dieser Untersuchung deutlich von 91,3 % auf 78,9 % senkt. Hatte bereits eine Immunsuppression zu einem beliebigen Zeitpunkt vor der stationären Aufnahme stattgefunden, wurde aufgrund der Beschwerdesymptomatik jedoch trotzdem eine 18F-FDG-PET/CT angefertigt, so blieb die Sensitivität hoch (92,3 %).

Dies lässt den Schluss zu, dass die 18F-FDG-PET/CT-Untersuchung für die Erstdiagnostik der PMR bei symptomatischen Patienten einen hohen diagnostischen Wert aufweist, unabhängig von einer bereits durchgeführten immunsuppressiven Medikation. Wird jedoch aufgrund der akuten Beschwerdesymptomatik unmittelbar vor der Untersuchung eine therapeutisch wirksame, entzündungshemmende, immunsuppressive Medikation angesetzt, so hat dies einen negativen Einfluss auf die Sensitivität dieser diagnostischen Methode.

Die im Folgenden diskutierten Publikationen untersuchten ebenfalls konkrete Anreicherungen in der 18F-FDG-PET/CT bei Patienten mit PMR.

Henckaerts et al. zeigten ebenfalls in einer prospektiven Untersuchung von 67 Patienten mit PMR im Vergleich zu 32 Nicht-PMR-Patienten einen signifikanten Unterschied zwischen beiden Patientengruppen in der skelettalen Anreicherung anhand eines Skeleton-Scores mit 12 Regionen. Es wurde ein optimaler Cut-off-Wert von 16 gefunden (Sensitivität 85,1 %, Spezifität 87,5 %) [12].

Unter Nutzung des visuellen Scoringsystems von Goerres et al. [9] wurden in der Publikation von Sondag et al. retrospektiv 50 PMR-Patienten mit 53 explizit nicht rheumatischen Patienten mit onkologischer Grunderkrankung an 17 skelettalen Regionen verglichen. Waren signifikante Anreicherungen an mindestens drei Regionen nachweisbar, lagen Sensitivität und Spezifität für die Diagnose PMR bei 74 % und 79 % respektive. Darüber hinaus zeigte sich, wie in unserer Studie, eine signifikant höhere Anreicherung bei 28 der PMR-Patienten, die keine Steroide bekamen, gegenüber 22 der PMR-Patienten, die steroidal vortherapiert waren [26].

Flaus et al. nutzen das Scoringsystem von Sondag et al. und empfahlen darüber hinaus, wie unsere Arbeit, die Nutzung einer Kombination zweier anatomischer Regionen für die Diagnose einer PMR. Sie verwendeten hierfür die interspinale Bursa sowie die Bursa trochanterica. Sensitivität und Spezifität lagen hier zwischen 73,2 und 78,6 % bzw. 87,5 und 80,1 % für die Diagnose PMR [7]. Emamifar et al. nutzten ebenfalls ein visuelles Scoringsystem, bestehend aus einer vierstufigen Analyse (0–3 Punkte) von 8 paarigen anatomischen Regionen. Diese erreicht eine Sensitivität von 79,7 % und eine Spezifität von 83,3 % bei einem Cut-off von 9 von 24 Punkten [6]. Kaneko et al. berichteten retrospektiv über 20 Patienten mit PMR, welche in der 18F-FDG-PET/CT signifikant höhere Anreicherungen an proximalen Gelenken wie Schultern und Hüften und extraartikulären Regionen wie Tuber ischiadicum, Processus spinosi und Bursa pertrochanterica zeigten [14]. Sie untersuchten die Anreicherungen innerhalb der PMR-Patientengruppe und im Vergleich zu distalen Gelenken, jedoch ohne Kontrollgruppe.

In einer aktuellen Vergleichsarbeit stellten van der Geest et al. die Methode von Henckaerts et al. als diejenige mit der höchsten Sensitivität (89,7 %) und Spezifität (84,2 %) für die Implementierung der Diagnose PMR heraus, die sich den Methoden von Sondag et al. und denjenigen von Owen und Flaus et al. überlegen zeigte [28].

Die Ergebnisse dieser Arbeiten konnten wir bestätigen, vor dem Hintergrund der Vielzahl an Herangehensweisen an die Diagnostik von PMR-Patienten in der 18F-FDG-PET/CT mittels unterschiedlicher Messpunkte erarbeiteten wir eine einfach anzuwendende Methode mit weiter gesteigerter Sensitivität und Spezifität. Diese nutzt die Kombination von vier SUVmax-Messwerten – beider anterolateraler Hüftkapseln und beider Tubera ischiadica. Über eine zusätzliche Aufschlüsselung der PMR-Patienten in solche mit einer Erstdiagnose und solche mit einer bereits bestehenden Diagnose erreicht unsere Methode eine Sensitivität von 91,3 % und eine Spezifität von 97,6 %, bei einem Cut-off von 11,0 SUV. Damit bestätigte unsere Untersuchung die Wertigkeit der 18F-FDG-PET/CT für die Diagnostik von PMR-Patienten in einem großen Patientenkollektiv aus 94 PMR-Patienten und 164 rheumatologischen Kontrollen, unter Nutzung der objektiveren Messmethode der SUVmax-Messung.

### Abgrenzung zur rheumatoiden Arthritis

Im Unterschied zur PMR, bei der sich Entzündungen der Synovia und periartikuläre Auffälligkeiten (Enthesitis) zeigen, präsentiert sich die RA im Ultraschall eher mit intraartikulären Auffälligkeiten [23]. Einer retrospektiven Arbeit mit 16 PMR-Patienten und 16 RA-Patienten von Yamashita et al. zufolge, die die Differentialdiagnostik beider Erkrankungen in der 18F-FDG-PET/CT thematisiert, erschwert dieser Unterschied in jedoch etwa identischer Lokalisation deren Differenzierung an den großen Gelenken mittels 18F-FDG-PET/CT [31]. Wir konnten die Ergebnisse von Yamashita et al. zum Teil bestätigen. Unsere Analyse an den Hüften demaskierte jedoch auch dort einen signifikanten Unterschied zwischen beiden Patientengruppen, genauer auch an der anterolateralen Hüftkapsel. Dies widerlegt den Ansatz von Yamashita et al. und zeigt, dass Patienten mit RA zwar an den Schultern eine Anreicherung ähnlich der von PMR-Patienten aufweisen, allerdings an den Hüften signifikant weniger stark anreichern und sich dort somit eine zusätzliche Möglichkeit der Differenzierung bietet.

In unserer detaillierten Analyse der Subgruppen der PMR- und der RA-Patienten zeigte sich, dass die 18F-FDG-PET/CT-Untersuchung zum Zeitpunkt einer Erstdiagnose im untherapierten Zustand für eine Differenzierung der PMR von einer RA geeignet ist, und zwar an den Messpunkten der gesamten Hüften, genauer der anterolateralen Hüftkapsel sowie den Sitzbeinen. Fand die Untersuchung zu einem späteren Zeitpunkt statt, so ließen sich diese beiden Krankheitsbilder durch eine 18F-FDG-PET/CT nicht mehr voneinander unterscheiden wie von Yamashita et al. vorbeschrieben.

### Overlap Riesenzellarteriitis/PMR

Allgemein anerkannt ist eine vaskulitische Komponente der PMR als Überlappen dieser Erkrankung mit der RZA. So zeigte etwa ein Drittel der Patienten mit PMR in einer prospektiven Studie von Blockmans et al. eine allgemeine vaskulitische Beteiligung [3]. Ein Überlappen der beiden rheumatischen Erkrankungen PMR und RZA wird in der Literatur mit einem Anteil von 16–21 % für den Anteil der PMR-Patienten, der zusätzlich an RZA erkrankt ist, angegeben. Umgekehrt liegt der Anteil der RZA-Patienten, die zusätzlich an PMR erkranken, bei 40–60 % [10, 11, 24]. In unserem Patientenkollektiv fand sich in einem Beobachtungszeitraum von über 44 Monaten mit 38 % ein vergleichbarer Anteil für eine PMR bei RZA, jedoch mit 9 % im literarischen Vergleich ein deutlich geringerer Anteil für eine RZA bei PMR. Letzteres kann darin begründet liegen, dass in dieser Arbeit ausschließlich stationäre, also schwerer erkrankte Patienten untersucht wurden. Patienten mit der Diagnose PMR, die ambulant versorgt wurden, finden hier keine Berücksichtigung.

Der Anteil der RZA-Patienten mit begleitender PMR jedoch scheint einen repräsentativen Wert anzugeben, da Patienten mit dem Verdacht auf eine Vaskulitis der großen Gefäße (RZA) in der Klinik für Rheumatologie des Johannes Wesling Klinikums Minden häufig eine 18F-FDG-PET/CT-Untersuchung erhalten.

### Diagnostischer Zusatznutzen der 18F-FDG-PET/CT

Trotz der nicht unerheblichen Strahlenexposition einer 18F-FDG-PET/CT überwiegt als rechtfertigende Indikation zu einer Fokussuche der Beschwerden der Patienten nicht nur der Ausschluss einer möglichen Paraneoplasie, sondern auch der diagnostische Zusatznutzen der 18F-FDG-PET/CT, vor dem Hintergrund, dass sich der Diagnose PMR eine Therapie mit erheblicher medikamentöser Immunsuppression anschließt.

In unserem Patientenkollektiv fand in 82 % der Fälle eine Ausschlussdiagnostik eines Malignoms bzw. einer Vaskulitis der großen Gefäße statt. In 4 % (*n* = 10) der Untersuchungen kam ein Malignom zur Darstellung, davon fünf Lymphome, drei pulmonale Malignome, eine diffuse Metastasierung mit unklarem Primarius, sowie ein Rezidiv eines Mammakarzinoms. Auch Patienten mit PMR sind von malignen Erkrankungen betroffen [29]. In unserem Kollektiv präsentierten von 97 Patienten mit PMR zwei Patienten eine zusätzliche maligne Erkrankung (multiples Myelom, chronische lymphatische Leukämie). Sander et al. publizierten 2006 eine Arbeit zur Häufigkeit okkulter Malignome als Ursache einer rheumatologischen Systemerkrankung. Sie stellten bei 8 % (67 von etwa 900 Patienten) der stationär im Zeitraum von 2002 bis 2005 in einer universitären Rheumatologieabteilung behandelten Patienten ein Tumorleiden fest [25]. In einer aktuell veröffentlichten Studie sahen Camellino et al. die PET/CT-Bildgebung als einzige Möglichkeit, eine Vaskulitis der großen Gefäße zu evaluieren [4].

Die 18F-FDG-PET/CT-Untersuchung bietet somit über die PMR-Diagnostik hinaus einen zusätzlichen Wert für die untersuchten Patienten.

### Limitationen

Eine Limitation unserer Arbeit ist zunächst ihr retrospektiver Charakter. Weiterhin zeigt die erstdiagnostizierte PMR-Subgruppe mit immunsuppressiver Medikation im stationären Aufenthalt unmittelbar vor der Untersuchung ein breiteres Konfidenzintervall, was auf eine große Varianz in der Stichprobe schließen lässt, sodass sowohl Sensitivität als auch Spezifität bei einer anderen Stichprobe anders ausfallen könnten. Die Gruppengröße der erstdiagnostizierten und prästationär vortherapierten PMR-Subgruppe ist kleiner, sodass die Cut-off-Werte in weiteren Studien untersucht werden sollten.

## Zusammenfassung

Unsere Untersuchung an einem großen Kollektiv zeigt, dass die 18F-FDG-PET/CT-Untersuchung als neue Methode für die Diagnose einer PMR mit einer hohen Sensitivität und Spezifität eingesetzt werden kann. Neben der hier beschriebenen Auswahl der optimalen Messpunkte ist die immunsuppressive Vorbehandlung von entscheidendem Einfluss auf die Wertigkeit der Resultate. Wenn Patienten mit einer klinischen Symptomkonstellation, die an eine PMR denken lässt, eine 18F-FDG-PET/CT-Untersuchung erhalten sollen, ist diese vor Therapiebeginn durchzuführen.

Die 18F-FDG-PET/CT-Untersuchung ermöglicht nicht nur den Ausschluss einer RZA und einer Paraneoplasie, auch erlaubt sie, mit hoher diagnostischer Genauigkeit die Erkrankung PMR zu erkennen und zusätzlich von einer RA abgrenzen zu können. Hierbei sollte ein besonderes Augenmerk auf der Region der anterolateralen Hüftkapsel, sowie dem Ursprung der ischiocruralen Muskulatur am Tuber ischiadicum liegen, da die Summe der Addition dieser vier Werte eine außergewöhnlich genaue Methode darstellt, Patienten mit PMR, die noch keine immunsuppressive Therapie erhalten haben, zu diagnostizieren (Sensitivität 91,3 %, Spezifität 97,6 %, Cut-off 11,0 SUV). Diese Herangehensweise ermöglicht die Früherkennung der PMR und erlaubt eine umgehende, zielgerichtete, adäquate immunsuppressive Therapie. Sie bietet eine Ergänzung zu den Kriterien der EULAR/ACR-Initiative. Zusammenfassend kann die 18F-FDG-PET/CT-Untersuchung als neues diagnostisches Kriterium für die Erkrankung PMR betrachtet werden.

Weitere Untersuchungen sollten angestrebt werden, um das Verfahren der 18F-FDG-PET/CT für Patienten mit PMR weiter zu evaluieren und zu optimieren.

## Fazit für die Praxis

- Stationäre Patienten mit klinischem Verdacht auf eine Polymyalgia Rheumatica (PMR) sollten vor Therapiebeginn eine 18-Fluordesoxyglukose-Positronenemissionstomographie/Computertomographie (18F-FDG-PET/CT) Untersuchung erhalten.
- Die Kombination der Regionen der anterolateralen Hüftkapsel und des Tuber ischiadicum bietet für PMR-Patienten bei Erstdiagnose und ohne vorherige Immunsuppression eine hohe diagnostische Aussagekraft.
- Eine 18F-FDG-PET/CT bei PMR-Patienten ermöglicht zusätzlich zur Diagnosefindung den Ausschluss einer Paraneoplasie und einer Riesenzellarteriitis.
